# Chemical characterization and antinociceptive and anti-inflammatory activities of the aqueous extract of *Myrciaria floribunda* (H. West ex Willd.) O. Berg fruit peel

**DOI:** 10.1007/s10787-026-02294-3

**Published:** 2026-06-12

**Authors:** Simone Patricia De Freitas Rosa, Izabelly Bianca da Silva Santos, Wendel César eSilva Pereira, Thaís Vitória Freitas de Souza, Paulo Henrique Eloi Fernandes, Gabriela Ribeiro de Sousa, José Fernandes da Silva Cardoso, Alisson Macário de Oliveira, Márcia Vanusa da Silva, Wêndeo Kennedy Costa, Maria Tereza dos Santos Correia

**Affiliations:** 1 Departamento de Bioquímica, Universidade Federal de Pernambuco, Recife, PE 50670-901 Brazil; 2 Departamento de Farmácia, Universidade Estadual da Paraíba, , Campina Grande, PB 58429-500 Brazil; 3 Departamento de Farmácia, Universidade Federal do Rio Grande do Norte, Natal, RN 59012-570 Brazil

**Keywords:** Caatinga, Cytokines, Inflammation, Myrtaceae, Pain

## Abstract

Natural products derived from medicinal plants represent an important source of bioactive compounds with potential therapeutic applications in the management of pain and inflammatory disorders. In this study, the chemical profile and pharmacological activities of the aqueous extract obtained from the fruit peel of *Myrciaria floribunda* (AeMf) were investigated. Chemical characterization was performed using high-performance liquid chromatography coupled with electrospray ionization mass spectrometry (HPLC–ESI–MSⁿ). The antinociceptive activity of AeMf was evaluated in mice using acetic acid–induced abdominal writhing, formalin, and tail immersion tests, while the anti-inflammatory potential was assessed through carrageenan-induced paw edema and peritonitis models. Chromatographic analysis revealed a chemical profile predominantly composed of organic acids and related metabolites, including quinic acid, citrate, maleate, and a fatty acid hexoside. In nociceptive models, AeMf produced a significant and dose-dependent reduction in acetic acid–induced writhing and markedly decreased paw-licking behavior in both phases of the formalin test. In addition, the extract increased tail withdrawal latency in the thermal nociception assay, indicating the involvement of both peripheral and central analgesic mechanisms. In inflammatory models, AeMf significantly inhibited carrageenan-induced paw edema, reduced leukocyte and neutrophil migration to the peritoneal cavity, and markedly suppressed the production of pro-inflammatory cytokines, including TNF-α and IL-1β. Collectively, these findings demonstrate that AeMf exerts potent antinociceptive and anti-inflammatory effects, likely mediated through the modulation of inflammatory mediators and cellular recruitment. The results highlight the therapeutic potential of *Myrciaria floribunda* as a promising natural source of bioactive compounds for the development of novel strategies for pain and inflammation management.

## Introduction

The plant family Myrtaceae represents one of the most diverse and widely distributed groups among angiosperms, occurring predominantly in tropical and subtropical regions, including Australia, South America, tropical areas of the Americas, and Southeast Asia. In Brazil, this family ranks among the most species-rich botanical groups, occupying the eighth position in terms of species diversity and encompassing numerous taxa of recognized ecological, economic, and pharmacological importance (Paulo Farias et al. 2020; Haro-González et al. 2021; Santos-Neves et al. 2024).

Within this family, the species *Myrciaria floribunda* (H. West ex Willd.) O. Berg, commonly referred to as cambuí, has attracted increasing scientific and commercial interest. Its fruits are widely appreciated for human consumption, either fresh or processed into various food products, including jams, distilled beverages, and other derived products. Additionally, cambuí fruits are frequently used in the preparation of juices, ice creams, liqueurs, preserves, and artisanal confectionery. These fruits also constitute a valuable source of pectic substances, which further enhances their technological and nutritional relevance (Santos et al. 2020; Santos de Moraes et al. 2023; Silva Santos et al. 2025).

Previous investigations have demonstrated that the fruits of *M. floribunda* exhibit distinctive physicochemical attributes that may vary according to the stage of ripening. These fruits are characterized by a high content of sugars, vitamin C, flavonoids, carotenoids, and phenolic compounds, which collectively contribute to their pronounced antioxidant capacity. Moreover, the essential oils obtained from different parts of the plant including leaves, flowers, and stems have been chemically characterized and shown to possess a range of biological activities, such as antimicrobial, antitumoral, and anticholinesterase effects (Oliveira et al. 2018a, b; Moraes et al. 2022; Santos et al. 2020; Costa et al. 2022).

Experimental studies involving the acetone extract derived from the bark of *M. floribunda* have demonstrated low acute oral toxicity, in addition to significant anti-inflammatory and antinociceptive activities (Santos et al. 2020). Likewise, the essential oil extracted from the leaves characterized by the presence of compounds such as δ-cadinene, bicyclogermacrene, α-cadinol, and epi-α-muurolol—has also exhibited pronounced anti-inflammatory and analgesic effects, supporting the therapeutic potential traditionally attributed to this species (Santos de Moraes et al. 2023).

Despite its recognized ethnopharmacological relevance and the growing scientific interest in its biological properties, comprehensive investigations addressing the bioactive constituents of *M. floribunda* fruits remain limited. Studies focusing on compounds obtained through aqueous extraction and their potential pharmacological activities, including anti-inflammatory and antinociceptive effects, are still scarce. In this regard, the remarkable biodiversity of the Caatinga biome an ecosystem with considerable yet underexplored biotechnological potential represents a promising source of novel bioactive compounds. Therefore, the investigation of native species such as *M. floribunda* may contribute to the identification of natural molecules with relevant therapeutic and biotechnological applications (Santos et al. 2020; Barbosa et al. 2021; Santos de Moraes et al. 2023).

Although previous studies have demonstrated the antinociceptive and anti-inflammatory properties of extracts obtained from *M. floribunda*, important gaps remain regarding the pharmacological potential of aqueous extracts obtained specifically from fruit peels and their effects on inflammatory mediators and leukocyte migration. Furthermore, studies integrating chemical characterization by HPLC–ESI–MSⁿ with in vivo inflammatory models and cytokine quantification are still limited.

## Materials and methods

### Plant material and preparation of the aqueous extract

Fruits of *M. floribunda* were collected in the Serra dos Paus Dóias region (Exu, Pernambuco, Brazil; 7°21′16.3″ S, 39°53′15.5″ W). Botanical identification was performed, and a voucher specimen (No. 92722) was deposited at the Dárdano de Andrade Lima Herbarium, Agronomic Institute of Pernambuco (IPA/PE).

The collected fruits were thoroughly washed with distilled water and subsequently dried at 36 °C for 48 h. After drying, the material was ground, and the fruit peel was separated for extraction. The aqueous extract was prepared by turbólise-assisted extraction, consisting of four cycles of 30 s, each followed by a 4 min interval, using a 10% (w/v) ratio (200 g of powdered peel in 2 L of distilled water). Following extraction, the resulting solution was frozen and subjected to lyophilization, yielding the dried extract, hereafter referred to as AeMf (*Myrciaria floribunda* Aqueous Extract).

### Chemical characterization

The chemical profile of AeMf was determined by high-performance liquid chromatography coupled with electrospray ionization tandem mass spectrometry (HPLC–ESI–MSⁿ), using a Shimadzu® chromatographic system hyphenated to an Ion Trap AmazonX mass spectrometer (Bruker). Chromatographic separation was achieved on a C18 reversed-phase column (250 × 4.6 mm, 5 µm particle size). The mobile phase consisted of water acidified with 0.1% formic acid (solvent A) and methanol (solvent B), employing a gradient elution from 5 to 100% B over 95 min, at a flow rate of 0.6 mL/min.

The sample was prepared at a concentration of 1 mg/mL in methanol, followed by filtration through a 0.45 µm PVDF membrane. An injection volume of 10 µL was used for analysis. UV detection was carried out in the range of 240–400 nm, with chromatograms recorded at 254, 280, 320, and 360 nm. Mass spectrometric analysis was performed under electrospray ionization (ESI) in negative mode, within an m/z range of 50–3000. The ionization parameters were set as follows: capillary voltage of 4.5 kV, nebulizing gas pressure of 35 psi, nitrogen flow rate of 8 mL/min, and source temperature of 300 °C. Spectral acquisition was performed at 2 s intervals. Structural elucidation of the detected compounds was based on MS data and MSⁿ fragmentation patterns.

### In Vivo experiments

All experimental procedures were conducted in accordance with the ethical guidelines for the use of laboratory animals and were approved by the Animal Ethics Committee of the Federal University of Pernambuco (approval no. 0029/2024). Male Swiss mice were obtained from the animal facility of the Keizo Asami Immunopathology Institute (ILIKA), Federal University of Pernambuco. The animals weighed between 35 and 40 g and were 8–10 weeks old. They were housed under controlled environmental conditions, including a 12 h light/dark cycle, temperature maintained at 22 ± 2 °C, and relative humidity of 50–55%. Standard laboratory food and water were provided ad libitum throughout the experimental period.

### Antinociceptive activity

For each experimental model, male mice were randomly assigned to six groups (n = 6 per group). The animals received the following treatments: AeMf (25, 50, or 100 mg/kg, orally), saline solution (0.9%, orally), morphine (10 mg/kg, intraperitoneally), or indomethacin (20 mg/kg, intraperitoneally). The aqueous extract and saline solution were administered 1 h prior to the beginning of the nociceptive assays, whereas morphine and indomethacin were administered 30 min before the tests. The selected doses were based on preliminary screening experiments and previous studies involving *Myrciaria* species and related plant extracts demonstrating antinociceptive and anti-inflammatory activities.

#### Acetic acid-induced abdominal writhing test

The writhing response was induced by intraperitoneal injection of acetic acid solution (0.85% v/v). Following administration, the animals were individually placed in transparent polyethylene chambers, and the number of abdominal constrictions (writhes) was recorded during the period from 5 to 15 min after acetic acid injection (Oliveira et al. 2018a, b).

#### Formalin-induced nociception test

In the formalin test, animals received an injection of 20 μL of formalin solution (2.5%, v/v) into the subplantar region of the right hind paw. The nociceptive response was quantified by measuring the time spent licking or biting the injected paw. Two distinct phases of nociceptive behavior were evaluated: the first phase (neurogenic pain), corresponding to the first 5 min after formalin administration, and the second phase (inflammatory pain), corresponding to the period between 15 and 30 min after injection (Hunskaar and Hole 1987).

#### Tail immersion test

Animals were pre-screened 24 h prior to the experiment by immersing their tails in warm water (55 ± 1 °C). Mice that maintained their tails immersed for longer than 5 s without withdrawal were excluded from the study. The remaining animals were randomly allocated to experimental groups. After 24 h, the animals received their respective treatments, and the distal portion of the tail was immersed in warm water at predetermined time points (0, 30, 60, 90, and 120 min after treatment). The latency time required for the animal to exhibit a nociceptive response (tail withdrawal) was recorded. A cutoff time of 20 s was established to prevent tissue damage (Khatun et al. 2015).

### Anti-inflammatory activity

Male mice were randomly distributed into five experimental groups (n = 6 per group) and treated with AeMf (25, 50, or 100 mg/kg, orally), saline solution (0.9%, orally), or indomethacin (20 mg/kg, intraperitoneally). The aqueous extract and saline solution were administered 1 h prior to the induction of inflammation, whereas indomethacin was administered 30 min before the experimental procedures.

#### Carrageenan-induced paw edema

Paw edema was evaluated using the carrageenan-induced inflammation model. Initially, the basal volume of the hind paws was measured using a digital caliper. After baseline measurements, animals received the respective treatments. One hour after treatment administration, inflammation was induced by subplantar injection of carrageenan solution (2%, 15 μL) into the right hind paw. Paw thickness was measured at 15, 30, 60, and 120 min following carrageenan administration. The degree of edema was calculated as the difference between the initial paw measurement and the values obtained at each experimental time point (Lapa et al. 2008).

#### Carrageenan-induced peritonitis

Inflammatory cell migration was assessed using a carrageenan-induced peritonitis model. Animals received an intraperitoneal injection of carrageenan solution (1%). After 4 h, the animals were euthanized, and 2 mL of heparinized phosphate-buffered saline (PBS) was injected into the peritoneal cavity. The peritoneal exudate was then carefully collected.

Total leukocyte counts were determined from the recovered peritoneal lavage fluid and expressed as percentages (Lapa et al. 2008). Total leukocyte counts were determined using an automated hematology analyzer. Differential leukocyte counts were performed by cytospin preparation of the peritoneal exudate, followed by Panoptic staining and analysis under light microscopy. In addition, the concentrations of pro-inflammatory cytokines, including TNF-α and IL-1β, were quantified in the peritoneal fluid according to the manufacturer’s instructions.

### Statistical analysis

All data were analyzed using GraphPad Prism® software (version 8.0). Results are expressed as mean ± standard deviation (SD). Statistical significance among experimental groups was determined using one-way analysis of variance (ANOVA), followed by Bonferroni or Dunnett’s post hoc tests when appropriate. Data from experiments involving repeated measurements over time (tail immersion and carrageenan-induced paw edema) were analyzed using two-way ANOVA followed by Bonferroni’s post hoc test, considering treatment and time as independent factors. Differences were considered statistically significant when *p* < 0.001.

## Results and discussion

The chemical characterization of the aqueous extract obtained from the peel of *M. floribunda* fruits (AeMf) was performed using high-performance liquid chromatography coupled with mass spectrometry (HPLC–MS) operating with electrospray ionization in negative mode (ESI⁻).

**Fig. 1:**
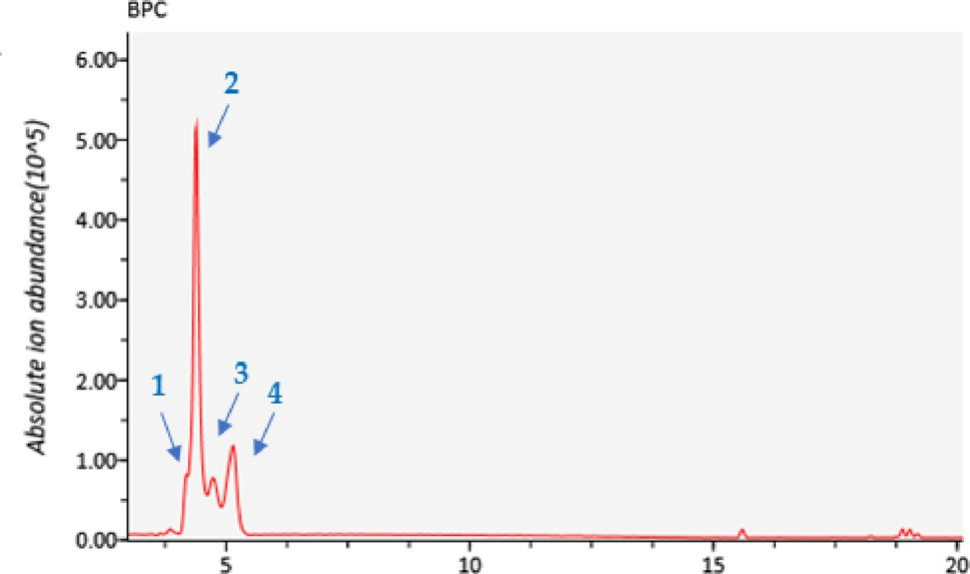
HPLC–MS analysis of the aqueous extract obtained from the fruit peel of *Myrciaria floribunda* (AeMf). Base peak chromatogram (BPC) showing the main peaks identified in negative ionization mode. Peaks 1–4 correspond to quinic acid (Rt 4.30 min), 6:3 + 6O fatty acyl hexoside (Rt 4.39 min), maleic acid (Rt 4.54 min), and citrate (Rt 5.14 min), respectively

The base peak chromatogram (BPC) revealed the presence of four predominant peaks within a retention time window between approximately 4 and 6 min (Fig. 1). This chromatographic profile suggests a predominance of relatively polar metabolites, a feature consistent with compounds typically extracted using aqueous solvents. Such molecules frequently include phenolic derivatives, flavonoids, and other hydrophilic secondary metabolites commonly reported in species of the Myrtaceae family.

Based on accurate mass measurements and MSⁿ fragmentation patterns, four major metabolites were tentatively identified in the AeMf (Table 1). These compounds were assigned as quinic acid (peak 1), a fatty acid hexoside (peak 2), maleic acid (peak 3), and citrate (peak 4). Overall, these metabolites belong predominantly to the class of organic acids and their derivatives, which are widely reported in species belonging to the Myrtaceae family. The chromatographic approach employed in the present study was primarily focused on the characterization of major polar constituents detectable under the selected analytical condition.

**Table 1:** Compounds identified in aqueous extract of *Myrciaria floribunda* (AeMf) fruits

Peak nº	Rt (min)	Mol. formula	Aducct	Mass (Da)	Error (ppm)	MS product ions	Putative identification	Chemical class
1	4.30	C_7_H_12_O_6_	[M-H]^−^	191.0551	− 1.007	127.0396, 109.0280, 93.0337, 87.0078, 85.0286	Quinic acid	Quinic acids and derivatives
2	4.39	C_12_H_18_O_12_	[M-H]^−^	353.0718	− 0.740	353.0716, 173.0086, 111.0080	6:3 + 6O fatty acyl hexoside	Fatty acyl glycosides of mono- and disaccharides
3	4.54	C_4_H_4_O_4_	[M-H]^−^	115.0028	− 0.885	115.0029, 71.0124	Maleic acid	Dicarboxylic acids and derivatives
4	5.14	C_6_H_8_O_7_	[M-H]^−^	191.0189	− 0.830	173.0082, 129.0195, 111.0078, 87.0079, 85.0287	Citrate	Tricarboxylic acids and derivatives

Quinic acid is an intermediate metabolite of the shikimate pathway and has been frequently associated with antioxidant and anti-inflammatory activities in plant-derived extracts (Choi et al. 2021; Benali et al. 2024; Li et al. 2024). Its presence in AeMf may contribute to the scavenging of reactive oxygen species and to the modulation of inflammatory mediators, mechanisms that have been reported for extracts enriched in this compound. Peak 2 was tentatively identified as a fatty acid hexoside, specifically a glycosylated lipid derivative (fatty acyl hexoside 6:3 with the addition of six oxygen atoms). The detection of this compound suggests the occurrence of lipid–sugar conjugates, which are known to participate in cellular signaling processes and may exhibit relevant biological activities, including potential antinociceptive effects.

In addition, the dicarboxylic and tricarboxylic acids detected maleic acid and citric acid are low molecular weight metabolites involved in central metabolic pathways such as the tricarboxylic acid cycle (Krebs cycle). Beyond their metabolic roles, these organic acids have also been associated with metal-chelating properties and the modulation of inflammatory responses (Amato et al. 2020; Jeong et al. 2022; Risanto et al. 2023; Książek 2023; Vliet et al. 2023; Hu et al. 2024). For instance, citric acid has been reported to exhibit antioxidant and metal-chelating properties, which may contribute to the overall biological potential of AeMf.

Taken together, the chemical profile obtained for AeMf indicates a predominance of hydrophilic metabolites, particularly organic acids and related derivatives, which is consistent with the polarity of the extraction solvent used. These compounds are widely recognized for their pharmacological relevance, especially regarding anti-inflammatory and analgesic properties (Jeong et al. 2022; Benali et al. 2024; Li et al. 2024). Therefore, the occurrence of these metabolites in AeMf provides a plausible chemical basis for the antinociceptive and anti-inflammatory effects observed in the biological assays, suggesting that the pharmacological activity may result from a synergistic interaction among the identified constituents.

**Fig. 2:**
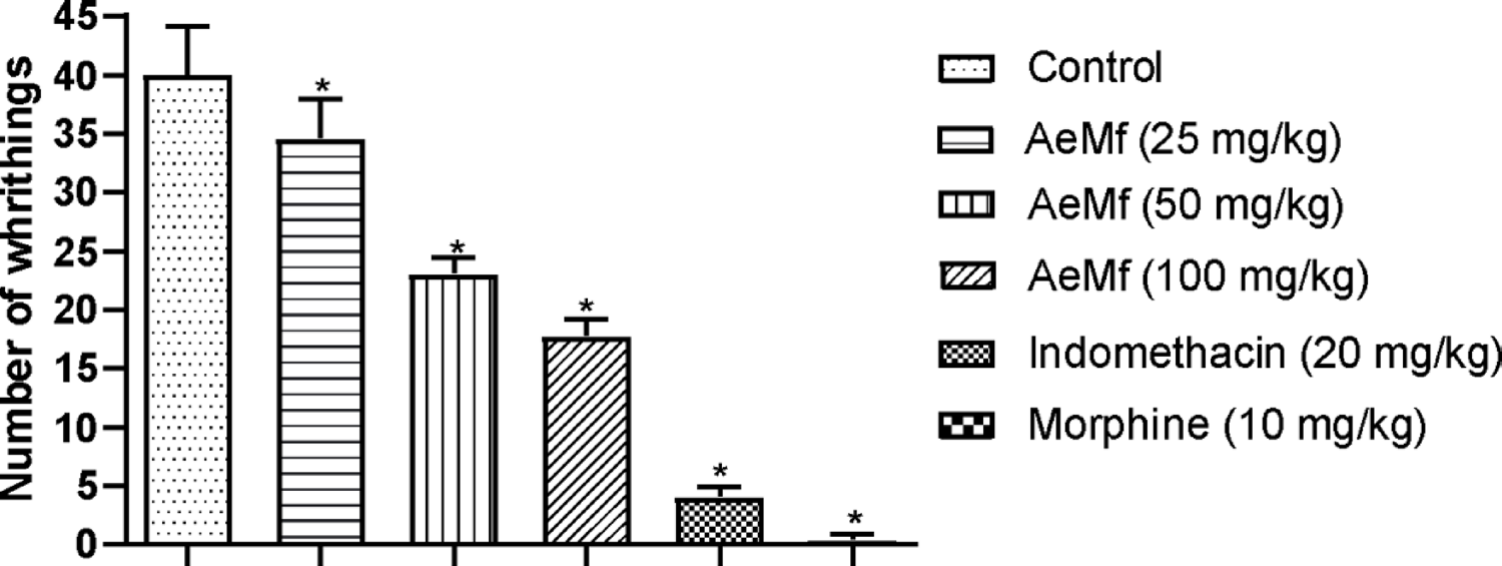
Antinociceptive effect of the aqueous extract obtained from *Myrciaria floribunda* fruits peel (AeMf) on acetic acid–induced abdominal writhing in mice. Data are expressed as mean ± SEM. *p* < 0.001 compared with the control group, as determined by one-way analysis of variance (ANOVA) followed by Dunnett’s post hoc test

Regarding the biological assays, treatment with different concentrations of AeMf significantly reduced the number of abdominal writhes induced by acetic acid in mice. Animals treated with AeMf at doses of 25, 50, and 100 mg/kg exhibited significant reductions (*p* < 0.001) in the number of writhing responses, corresponding to inhibition rates of 42.5%, 55.85%, and 90.00%, respectively. The group treated with morphine exhibited the highest inhibitory effect, showing a reduction of 99.17% in the number of writhes, while the indomethacin-treated group demonstrated a 90.00% reduction when compared with the control group (Fig. 2).

The ability of AeMf to modulate inflammatory and peripheral nociception was clearly demonstrated in the acetic acid–induced abdominal writhing model. This assay is widely recognized as a classical experimental model of visceral inflammatory pain. Intraperitoneal administration of acetic acid triggers the release of a cascade of endogenous inflammatory mediators within the peritoneal cavity, including bradykinin, substance P, and pro-inflammatory cytokines such as tumor necrosis factor alpha (TNF-α) and interleukin-1 beta (IL-1β). These mediators directly activate and sensitize peripheral nociceptors, resulting in the characteristic writhing behavior observed in rodents (Oliveira et al. 2018a, b; Costa et al. 2022).

Treatment with AeMf produced a marked and dose-dependent reduction in the number of abdominal writhes, reaching an inhibition of 90.00% at the highest tested dose (100 mg/kg). Notably, this effect was statistically comparable to that observed with indomethacin (90.00%), a nonsteroidal anti-inflammatory drug (NSAID) whose primary mechanism of action involves the non-selective inhibition of cyclooxygenase (COX) enzymes, thereby suppressing prostaglandin (PG) biosynthesis (Lin et al. 2023).

The strong antinociceptive activity observed for AeMf at 100 mg/kg, comparable to that produced by indomethacin, suggests that the extract may exert its effects—at least in part—through modulation of the COX pathway and the consequent reduction of prostaglandin production. Since prostaglandins are key mediators in the sensitization of peripheral nociceptors, their inhibition may account for the pronounced reduction in nociceptive behavior observed in the present study (Abdellatif et al. 2021).

Overall, these findings indicate that AeMf effectively attenuates inflammatory and peripheral pain. However, the acetic acid–induced writhing test presents limited specificity for distinguishing central and peripheral mechanisms of analgesia. Therefore, to further clarify the antinociceptive potential of AeMf, additional experimental models were employed, including the formalin test and the tail immersion test.

In the formalin assay, oral administration of AeMf (25, 50, and 100 mg/kg) significantly reduced (*p* < 0.001) the licking time of the injected paw during the first phase (neurogenic phase) by 37.33%, 48.34%, and 48.62%, respectively, when compared with the control group. In contrast, morphine (10 mg/kg) produced a pronounced reduction of 90.08%, while indomethacin reduced nociceptive behavior by 31.27% relative to the control group.

**Fig. 3:**
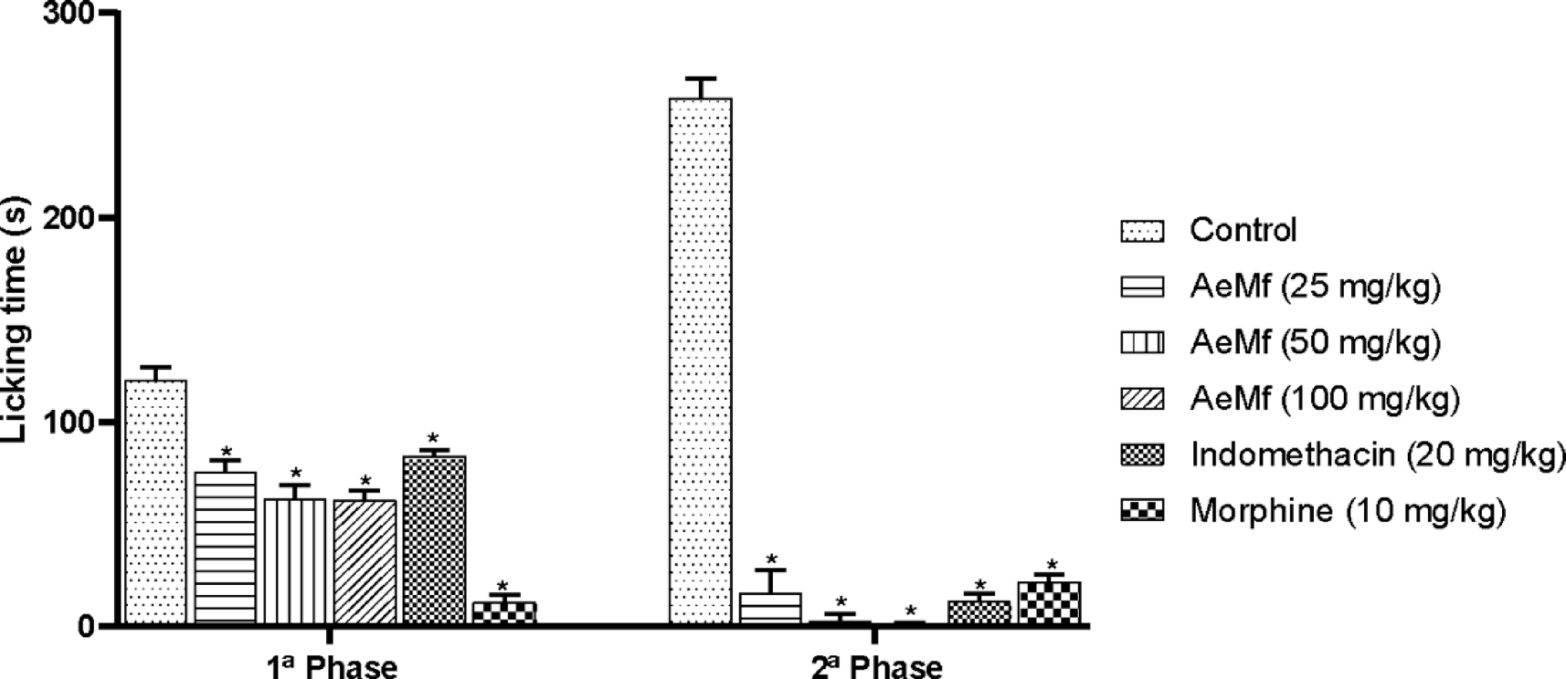
Antinociceptive effects of the aqueous extract obtained from *Myrciaria floribunda* fruits peel (AeMf) during both phases of the formalin test. Data are presented as mean ± SEM. *p* < 0.001 compared with the control group, as determined by one-way ANOVA followed by Dunnett’s post hoc test

During the second phase of the formalin test (inflammatory phase), AeMf exhibited an even more pronounced effect. Treatment with the extract at doses of 25, 50, and 100 mg/kg resulted in reductions of paw-licking time by 93.67%, 98.83%, and 100.00%, respectively. The positive controls, indomethacin (20 mg/kg) and morphine (10 mg/kg), also produced substantial inhibition, reducing the nociceptive response by 95.09% and 91.61%, respectively, when compared with the control group (Fig. 3).

The results obtained in the formalin assay further support the pharmacological activity of AeMf and provide additional insights into its possible mechanism of action (Bannon et al. 2007; Rahimi et al. 2023). The significant reduction in paw-licking time observed during the first phase (neurogenic phase), which is primarily mediated by the direct activation of nociceptors particularly through TRPA1 channels suggests the involvement of central analgesic mechanisms, potentially related to modulation of spinal or supraspinal neurotransmission pathways (Fischer et al. 2014; Liu et al. 2021). Notably, the efficacy of AeMf in this phase (48.62%) exceeded that observed for indomethacin (31.27%), a classical nonsteroidal anti-inflammatory drug (NSAID), suggesting that the extract may exert analgesic effects beyond those associated solely with peripheral anti-inflammatory activity.

The second phase of the formalin test, typically occurring between 15 and 30 min after formalin injection, is characterized by a localized inflammatory response within the paw tissue. This phase involves the release of several inflammatory mediators, including histamine, serotonin, bradykinin, and prostaglandins. In addition to directly activating nociceptors, these mediators contribute to the development of central sensitization within the dorsal horn of the spinal cord, resulting in increased pain sensitivity (Costa et al. 2022).

One of the most notable findings of this study was the remarkable inhibition of the inflammatory phase by AeMf. At the highest tested dose (100 mg/kg), the extract completely suppressed the nociceptive response, reaching an inhibition rate of 100.00%. Importantly, under these conditions, the antinociceptive effect of AeMf exceeded that observed for the positive controls, including indomethacin (95.09%) and the opioid analgesic morphine (91.61%). These findings indicate that AeMf possesses potent peripheral anti-inflammatory properties, which appear to play a crucial role in its overall analgesic profile. The ability of the extract to almost completely abolish inflammatory nociception suggests a highly efficient interference with the production or activity of inflammatory mediators at the site of tissue injury, possibly involving mechanisms beyond simple inhibition of the cyclooxygenase pathway (Moraes et al. 2022; Silva Santos et al. 2025).

Taken together, these findings demonstrate that AeMf exerts significant effects in both phases of the formalin test, indicating its potential to alleviate both neurogenic and inflammatory pain. To further investigate the possible involvement of suggestive of possible central involvement in the analgesic activity of the extract, the tail immersion test was performed.

**Fig. 4:**
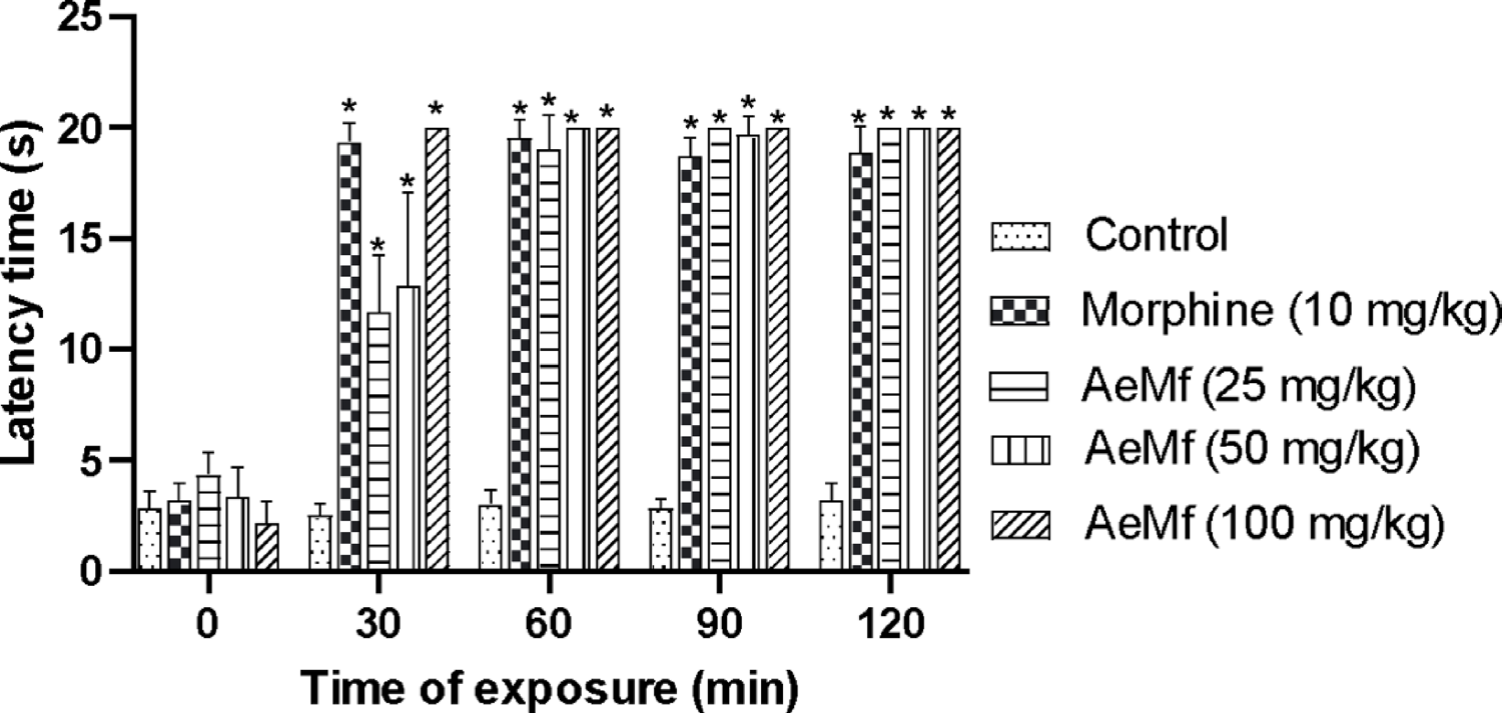
Antinociceptive effect of the aqueous extract obtained from *Myrciaria floribunda* fruits peel (AeMf) in the tail immersion test. Data are presented as mean ± SEM. *p* < 0.001 compared with the control group, as determined by two-way ANOVA followed by Bonferroni’s post hoc test

In this model of thermal nociception, animals treated with AeMf at all tested doses exhibited a significant increase in tail withdrawal latency compared with the control group starting at 30 min after treatment, with the antinociceptive effect persisting throughout the experimental period. Moreover, the responses observed for AeMf were comparable to those obtained with morphine after 60 min for all tested doses, reinforcing the hypothesis that the extract may also exert central analgesic effects (Fig. 4).

The antinociceptive profile of AeMf is not limited to peripheral anti-inflammatory effects. A more comprehensive analysis of the first phase of the formalin test in conjunction with the tail immersion assay reveals a broader pharmacological profile involving modulation of neurogenic pain and suggestive of possible central involvement. The first phase of the formalin test (0–5 min) reflects acute neurogenic pain resulting from the direct chemical activation of peripheral nociceptors, predominantly C and Aδ fibers. This process involves the release of neurotransmitters such as substance P and excitatory amino acids, including glutamate (Simões et al. 2017; Silva-Alves et al. 2021; Nguyen et al. 2025).

Treatment with AeMf significantly reduced paw-licking time during this phase, reaching an inhibition of up to 48.62% at the dose of 100 mg/kg. This finding indicates that the extract contains bioactive compounds capable of interfering with nociceptor activation or with the early transmission of nociceptive signals. Previous studies involving *M. floribunda* fruit peels suggested a possible involvement of the opioidergic system in the antinociceptive activity of this species (Silva Santos et al. 2020). Although opioid antagonists were not evaluated in the present study, the central antinociceptive effects observed in the tail immersion test may be consistent with this hypothesis.

A plausible molecular mechanism underlying this neurogenic effect may involve modulation of ion channels expressed in sensory neurons. Members of the transient receptor potential (TRP) channel family, particularly TRPV1 (capsaicin receptor) and TRPA1, act as key molecular sensors for noxious chemical stimuli, including formalin. Activation of these channels leads to nociceptor depolarization and subsequent generation of action potentials. Notably, the activity of these channels is highly sensitive to pH changes; for instance, acidic environments are well-established activators of TRPV1 channels (Kwon et al. 2009; Bölcskei et al. 2010; Louis-Gray et al. 2022; Ivanova et al. 2024).

Considering that the chemical composition of AeMf is dominated by organic acids such as quinic, maleic, and citric acids it is reasonable to hypothesize that these compounds may modulate TRP channel activity. Such modulation could involve an initial activation followed by rapid desensitization, a mechanism described for several TRP agonists, ultimately leading to reduced nociceptor excitability and attenuation of acute neurogenic pain observed in the first phase of the formalin test (Rios et al. 2013; Argôlo et al. 2022; Kumamoto 2024).

The most compelling evidence supporting a suggestion of possible central involvement of action for AeMf arises from the tail immersion test. This model evaluates responses to thermal nociceptive stimuli and is considered highly predictive of centrally acting analgesics, such as opioids, as it requires integration of nociceptive signals at supraspinal levels (Ramabadran et al. 1989; Zhou et al. 2014; Costa et al. 2022).

In this assay, AeMf significantly increased tail withdrawal latency at all tested doses, with the antinociceptive effect sustained throughout the experimental period. Importantly, the analgesic response produced by AeMf became comparable to that observed in the morphine-treated group after 60 min. This finding strongly suggests that one or more constituents of the extract—either individually or through synergistic interactions—may contribute to centrally mediated analgesic effects.

This central activity distinguishes AeMf from purely peripheral agents, such as most nonsteroidal anti-inflammatory drugs (NSAIDs), and aligns its pharmacological profile, at least in part, with that of centrally acting analgesics. Such a dual mechanism of action—encompassing both peripheral anti-inflammatory and central analgesic effects—enhances the therapeutic potential of AeMf and highlights its relevance as a candidate for the development of novel analgesic agents (Srebro et al. 2022; Silva et al. 2023; Jaffal et al. 2025).

Consistent with this interpretation, the significant reduction in acetic acid–induced abdominal writhing and the decreased paw-licking time observed during the second phase of the formalin test further support the anti-inflammatory activity of AeMf. Based on these findings, the anti-inflammatory potential of the extract was further investigated using carrageenan-induced models of paw edema and peritonitis.

**Fig. 5:**
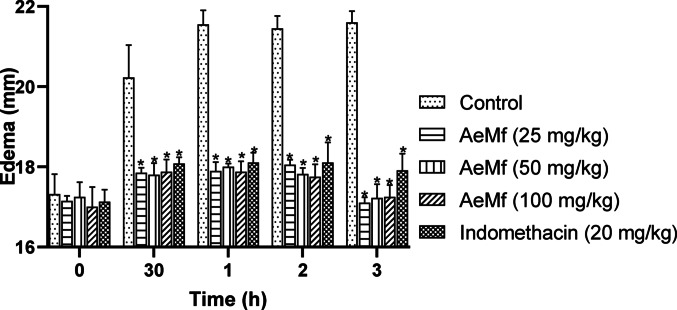
Anti-inflammatory effect of different concentrations of the aqueous extract obtained from *Myrciaria floribunda* fruits peel (AeMf) in the carrageenan-induced paw edema model. Data are expressed as mean ± SEM. *p* < 0.001 compared with the control group, as determined by two-way ANOVA followed by Bonferroni’s post hoc test

In the paw edema model, treatment with AeMf at doses of 25, 50, and 100 mg/kg significantly inhibited the increase in paw volume compared with the control group. This inhibitory effect was sustained throughout the experimental period and was comparable to that observed in the indomethacin-treated group, indicating a robust anti-inflammatory response (Fig. 5).

In the carrageenan-induced paw edema model, which mimics the vascular phase of acute inflammation, AeMf significantly and consistently inhibited the increase in paw volume. This experimental model is characterized by a biphasic inflammatory response: an early phase mediated primarily by the release of histamine and serotonin, followed by a late phase sustained mainly by the production of prostaglandins, nitric oxide, and pro-inflammatory cytokines (Hijazy et al. 2023; Renny et al. 2024).

The inhibitory effect observed for AeMf, with a profile comparable to that of indomethacin, supports the hypothesis that the extract may interfere with the synthesis and release of key mediators involved in the late phase of inflammation, particularly those associated with the cyclooxygenase (COX) pathway. These mediators play a critical role in increasing vascular permeability and promoting plasma extravasation into the interstitial space, ultimately leading to edema formation (Esmaili et al. 2021; Salem et al. 2021; Hijazy et al. 2023; Renny et al. 2024).

To further investigate the cellular component of the inflammatory response, the carrageenan-induced peritonitis model was employed, which allows quantification of leukocyte migration toward an inflammatory focus (Siqueira Patriota et al. 2022; Sousa-Neto et al. 2022). In this model, animals treated with AeMf (25, 50, or 100 mg/kg, orally) exhibited a significant reduction in total leukocyte migration to the peritoneal cavity, with inhibition rates of 39.50%, 61.72%, and 75.30%, respectively. Similarly, neutrophil recruitment was markedly suppressed, with reductions of 49.05%, 62.26%, and 77.35% for the same respective doses. In comparison, treatment with indomethacin resulted in a reduction of leukocyte migration by 79.01% and neutrophil migration by 79.24% (Table 2).

**Table 2:** Anti-inflammatory effect of the aqueous extract obtained from *Myrciaria floribunda* fruits peel (AeMf) on leukocyte and neutrophil migration in the peritoneal fluid of mice subjected to carrageenan-induced peritonitis

	Dose (mg/kg)	Leukocytes (10^5^/ml)	Inhibition (%)	Neutrophils (10^5^/ml)	Inhibition (%)
Control	–	8.1 ± 0.9	–	5.3 ± 0.5	–
indomethacin	20	1.7 ± 0.2*	79.01	1.1 ± 0.1*	79.24
AeMf	25	4.9 ± 0.3*	39.50	2.7 ± 0.2*	49.05
	50	3.1 ± 0.2*	61.72	2.0 ± 0.2*	62.26
	100	2.0 ± 0.2*	75.30	1.2 ± 0.1*	77.35

Data are presented as mean ± SEM. *p* < 0.001 compared with the control group, as determined by one-way ANOVA followed by Dunnett’s post hoc test

Treatment with AeMf produced a pronounced and dose-dependent inhibition of leukocyte chemotaxis into the peritoneal cavity. The extract reduced total leukocyte migration by up to 75.30% and, more specifically, neutrophil recruitment by up to 77.35%. Neutrophils represent the first line of immune cells recruited to inflammatory sites, where they play an essential role in pathogen elimination. However, their activation also contributes to tissue damage through the release of proteolytic enzymes and reactive oxygen species (Filep 2022; Wang et al. 2022; Herrero-Cervera et al. 2022). Therefore, the ability of the extract to markedly suppress neutrophil infiltration represents a critical anti-inflammatory mechanism (Silva Santos et al. 2025).

Leukocyte migration from the bloodstream into inflamed tissues is a highly coordinated process regulated by gradients of chemoattractant molecules. Pro-inflammatory cytokines released by resident macrophages following carrageenan stimulation act as key initiators of this process. These cytokines promote the expression of adhesion molecules, such as selectins and integrins, on endothelial cells and stimulate the production of chemokines that guide circulating leukocytes toward the inflammatory site (Singh et al. 2021; Sadeghi et al. 2023; Costa et al. 2024).

The results obtained in the present study demonstrate that AeMf strongly suppresses leukocyte recruitment. This finding suggests a direct mechanistic link between the observed reduction in inflammatory cell infiltration and the extract’s capacity to interfere with upstream signaling pathways responsible for cytokine-mediated leukocyte trafficking (Moraes et al. 2022). Thus, AeMf appears not only to attenuate the final manifestations of inflammation, such as edema formation, but also to interfere with early cellular and molecular events that drive the propagation of inflammatory responses.

**Fig. 6:**
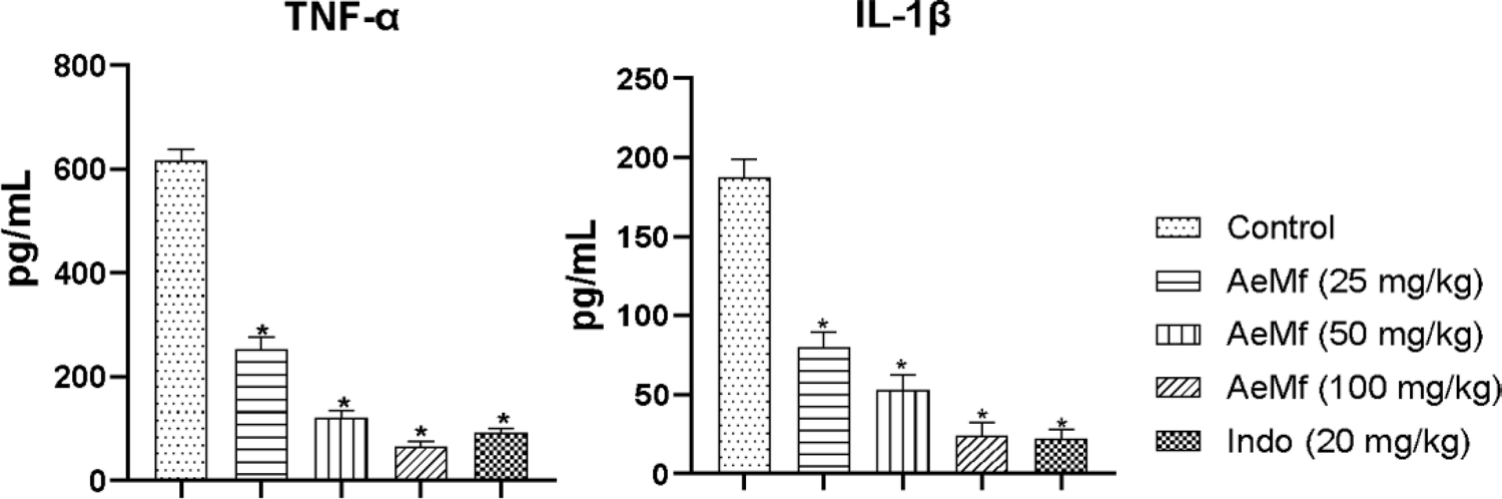
Anti-inflammatory effect of the aqueous extract obtained from *Myrciaria floribunda* fruits peel (AeMf) on TNF-α and IL-1β levels in the peritoneal fluid following carrageenan-induced inflammation. Data are presented as mean ± SEM. *p* < 0.001 compared with the control group, as determined by one-way ANOVA followed by Dunnett’s post hoc test

Consistent with this interpretation, treatment with the aqueous extract obtained from the fruits of *M. floribunda* significantly reduced the production of pro-inflammatory cytokines in the peritoneal fluid. At all tested doses (25, 50, and 100 mg/kg, orally), AeMf markedly decreased the levels of tumor necrosis factor alpha (TNF-α) by 57.2%, 71.74%, and 87.08%, respectively, and interleukin-1 beta (IL-1β) by 58.99%, 80.56%, and 89.26%, respectively, when compared with the control group. For comparison, indomethacin reduced TNF-α levels by 88.18% and IL-1β levels by 85.05%. The ability of AeMf to substantially suppress the production of these key pro-inflammatory cytokines further supports its potent anti-inflammatory activity and provides additional mechanistic evidence for the modulation of inflammatory signaling pathways (Fig. 6).

The efficacy observed at the highest dose of AeMf (100 mg/kg) was notably comparable to that of indomethacin, producing inhibition rates of 88.18% for TNF-α and 85.05% for IL-1β. These cytokines are widely recognized as key upstream regulators of the inflammatory cascade. Once released, TNF-α and IL-1β trigger the transcription of a broad repertoire of pro-inflammatory genes, leading to the production of secondary mediators, including additional cytokines and chemokines, adhesion molecules, and critical inflammatory enzymes such as cyclooxygenase-2 (COX-2) and inducible nitric oxide synthase (iNOS) (Oh et al. 2022; Rong et al. 2022; Caronni et al. 2023; Fok et al. 2024).

By markedly suppressing the production of these two central cytokines, AeMf appears to act at an early and pivotal checkpoint of the inflammatory response. Such an effect suggests a mechanism that extends beyond the classical inhibition of the cyclooxygenase pathway. Rather than targeting a single enzymatic step, the extract seems capable of modulating upstream signaling events that orchestrate the inflammatory cascade.

This ability to regulate cytokine production indicates that AeMf may possess significant immunomodulatory potential, contributing to the restoration of immune homeostasis rather than merely blocking isolated inflammatory mediators. From a pharmacological perspective, such a mechanism is particularly attractive for the development of novel anti-inflammatory therapies, as it may provide a broader therapeutic effect while potentially minimizing the adverse effects commonly associated with conventional nonsteroidal anti-inflammatory drugs (NSAIDs) (Jena et al. 2022; Thitinarongwate et al. 2022; El-Demerdash et al. 2025).

## Conclusion

The AeMf extract presented a chemical profile dominated by organic acids and phenolic derivatives, including quinic acid, citrate, maleate, and a fatty acid hexoside, compounds recognized for their anti-inflammatory and antioxidant activities. The presence of these metabolites provides mechanistic support for the observed pharmacological effects, since AeMf demonstrated significant antinociceptive activity in peripheral and central pain models, with efficacy comparable to or superior to indomethacin and morphine in different experimental phases. Furthermore, the extract reduced edema and leukocyte migration in carrageenan-induced inflammatory models, indicating robust anti-inflammatory action. Thus, the findings highlight that AeMf possesses analgesic and anti-inflammatory mechanisms, emphasizing it as a promising natural source of bioactive molecules for the management of pain and inflammation.

## Data Availability

No datasets were generated or analysed during the current study.
